# *Clostridioides difficile* and *Enterococci*’s Interplay in the Human Gut: Bacterial Alliance or Competition? A Systematic Literature Review

**DOI:** 10.3390/jcm12154997

**Published:** 2023-07-29

**Authors:** Guido Granata, Francesco Schiavone, Fabrizio Taglietti, Nicola Petrosillo

**Affiliations:** 1 Systemic and Immune Depression-Associated Infection Unit, National Institute for Infectious Diseases “L. Spallanzani”, IRCCS, 00149 Roma, Italy; fabrizio.taglietti@inmi.it; 2 Divers and Raiders Group Command “Teseo Tesei” COMSUBIN, Medical Service, Italian Navy, 19025 Portovenere, Italy; 3 Infection Prevention & Control-Infectious Disease Service, Fondazione Policlinico Universitario Campus Bio-Medico, 00127 Rome, Italy; n.petrosillo@policlinicocampus.it

**Keywords:** *Clostridioides difficile*, *Enterococcus* spp., colonization, vancomycin resistance, fidaxomicin, microbiota, bacterial interaction, pathogenesis, multidrug resistance, patients’ outcome

## Abstract

*Clostridioides difficile* and *Enterococcus* spp. are two common bacterial pathogens populating the human microbiota. We possess scant data on how *Clostridioides difficile* interacts with *Enterococcus* spp. in the gut microbiota in subjects colonized with *Clostridioides difficile* or during a *Clostridioides difficile* infection. We carried out a systematic review of studies on *Enterococcus* spp. and *Clostridioides difficile*’s interaction in the gut microbiota and on the effect of *Enterococcus* spp. gut colonization on CDI development. Studies on *Enterococcus* spp. and *Clostridioides difficile*’s interaction in the gut microbiota and on the effect of *Enterococcus* spp. gut colonization on CDI were searched using the search terms “clostridium”, “clostridioides”, “difficile” and “enterococcus” on the MEDLINE and SCOPUS databases. PubMed was searched until 1 May 2023. An English language restriction was applied. The risk of bias in the included studies was not assessed. Quantitative and qualitative information was summarized in textual descriptions. Fourteen studies, published from August 2012 to November 2022, on *Clostridioides difficile* and *Enterococcus* spp.’s interaction in the gut microbiota met the inclusion criteria. The studies included in our systematic review reported evidence that the *Enterococcus* spp. intestinal burden represents a risk factor for the occurrence of CDI. There is supporting evidence that *Enterococcus* spp. play a role in CDI development and clinical outcomes.

## 1. Introduction

In the last decade, our knowledge on the microbial ecosystem populating the human gut has considerably increased. The gut microbiota is an extremely heterogeneous environment populated by different bacteria, viruses and fungi continuously interacting with each other and with the human immune system. 

In the case of gut dysbiosis, *Clostridioides difficile* represents a potential pathogen causing diarrhea.

However, we possess scant data on how *Clostridioides difficile* interacts with the variety of microorganisms populating the microbiota in subjects colonized with *Clostridioides difficile* or in *Clostridioides difficile* infection (CDI) patients.

There is a lack of knowledge on the interaction between two common bacterial pathogens populating the human microbiota, i.e., *Clostridioides difficile* and *Enterococcus* spp. 

Interestingly, *Enterococcus* spp. and *Clostridioides difficile* share several similarities. Both of these two bacteria belong to the *Firmicutes* phylum and possess intrinsic resistance to common antibiotics [1,2,3,4,5]. Moreover, they may compose part of the gut microbiota in healthy subjects but are also among the main causes of opportunistic infections [1,2,3,4,5].

Moreover, *Enterococcus* spp. and *Clostridioides difficile* share the ability to proliferate following microbiota dysbiosis, i.e., after antibiotic administration [6,7]. Adding complexity to this picture, *Enterococcus* spp. possess the ability to acquire resistance to several antibiotics, including the first-line CDI antimicrobial treatment, vancomycin [8].

Recently, some studies investigated the potential interplay between *Enterococcus* spp. and *Clostridioides difficile* in the gut microbiota and the impact of *Enterococcus* spp. on CDI occurrence, development and clinical outcomes [8,9]. However, the effect of *Enterococcus* spp. gut colonization on susceptibility to CDI and on CDI patients’ outcomes remains largely unknown. 

We performed a systematic literature review to assess the available data on *Enterococcus* spp. and *Clostridioides difficile*’s interaction in the gut microbiota and on the effect of *Enterococcus* spp. gut colonization on CDI development.

## 2. Materials and Methods

### 2.1. Search Strategy and Article Identification

Published articles on *Enterococcus* spp. and *Clostridioides difficile*’s interaction in the gut microbiota and on the effect of *Enterococcus* spp. gut colonization on CDI were searched on the MEDLINE and SCOPUS databases.

Searching the MEDLINE database, the following search terms were used: [(clostridium) OR (clostridioides) AND (difficile) AND (enterococcus)]. The filters clinical study, clinical trial, meta-analysis, multicenter study, observational study and randomized controlled trial were applied with respect to article type, and the filter from 1 January 2020 to 1 May 2023 was applied with respect to publication date.

Searching the SCOPUS database, the following search terms were used: [(clostridioides) AND (difficile) AND (enterococcus)]. The filter article was applied with respect to document type; the filter English was applied with respect to language; the filter enterococcus was applied with respect to keywords; and the filter from 2019 to 2023 was applied with respect to publication date.

Supplementary Figure S1 describes the specifications of the query details used on the MEDLINE and SCOPUS databases, respectively (Figure S1).

No attempt was made to obtain information about unpublished studies. Review articles and meta-analyses, correction articles, case reports, editorials and clinical trial protocols were excluded from further assessment.

### 2.2. Eligibility Criteria

Original articles reporting data on *Clostridioides difficile* and *Enterococcus* spp.’s interaction in the gut microbiota were eligible for inclusion.

### 2.3. Study Selection and Data Extraction

Eligibility assessment and extraction of data were performed independently by two investigators. Each investigator was blinded to the other investigator’s data extraction. In the case of disagreement between the two reviewers, a third reviewer was consulted. The risk of bias in the included studies was not assessed.

For each included study, we collected data regarding the study design, the study population and the study setting, the sample size and the patients’ outcome if applicable.

### 2.4. Data Synthesis

Quantitative and qualitative information was summarized by means of textual descriptions.

## 3. Results

### 3.1. Study Description

**Figure 1:**
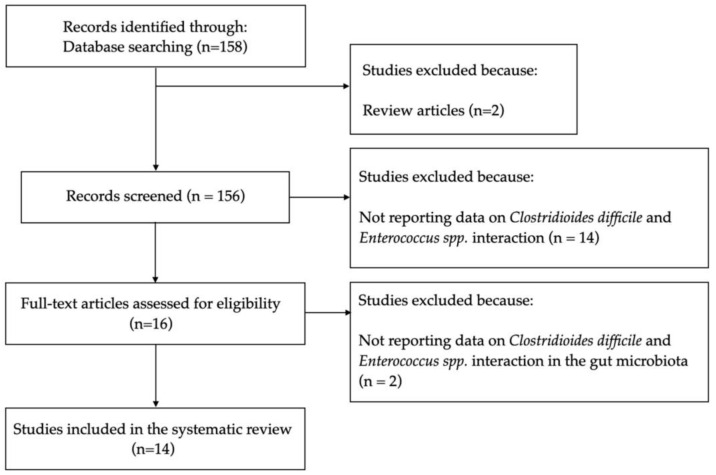
Selection process of the included studies.

Figure 1 shows the selection process of the included studies. Through literature searches, 158 studies were identified, which were published from November 1989 to November 2022.

Two studies were excluded because they were review articles. One hundred forty studies were excluded for not reporting data on *Clostridioides difficile* and *Enterococcus* spp.’s interaction.

From the remaining sixteen studies, two were excluded for not reporting data on *Clostridioides difficile* and *Enterococcus* spp.’s interaction in the gut microbiota [10,11]. Therefore, 14 studies were included in this systematic review (Figure 1) [1,12,13,14,15,16,17,18,19,20,21,22,23,24]. Of the 14 included studies, 3 studies evaluated *Enterococcus* spp. and *Clostridioides difficile*’s interplay during CDI [12,13,14], 4 studies evaluated the *Enterococcus* spp. intestinal burden as a risk factor for CDI onset [1,15,16,17], 3 studies reported the prevalence of vancomycin-resistant *Enterococcus* spp. in CDI patients [18,19,20] and 4 studies evaluated the effect of CDI antimicrobial treatment on the occurrence of vancomycin-resistant *Enterococcus* spp. colonization [21,22,23,24].

Among the included studies, seven were prospective or retrospective cohort studies [1,13,15,16,18,22,23], one was a case-control study [17], two were randomized phase III clinical trials [21,24], three were microbiological studies [14,19,20] and one was an in vivo study in the animal model [12].

Among the 14 included studies, 1 was an animal model study [12]; 1 study comprised two parts, the first one using the animal model and the second one being performed on pediatric human subjects [13]; 1 study enrolled pediatric patients [14]; and 11 studies enrolled adult patients [1,15,16,17,18,19,20,21,22,23,24]. Of the 13 studies performed on human subjects, 6 studies included less than 100 patients [13,14,17,19,20,21], whilst 7 studies included more than 100 patients [1,15,16,18,22,23,24].

A summary description of the included studies is reported in Table 1, Table 2, Table 3 and Table 4.

### 3.2. Enterococcus spp. and C. difficile’s Interplay during Clostridioides Difficile Infection

An in vivo study used the mouse model to test the hypothesis that specific bacterial gut communities determine a variation in CDI severity. Different gut communities were derived by colonizing germfree mice with human fecal communities. The mice were then infected with a *Clostridioides difficile* clinical isolate, resulting in morbidity and histopathologic differences. Fecal communities rich in *Enterococcus* spp. were associated with more severe CDI outcomes [12].

Moreover, a study aimed to define the interaction between *Enterococcus* spp. and *Clostridioides difficile* during CDI. The study comprised two parts; the first one was performed using the mouse model, and the second one was on pediatric human patients to evaluate the role of *Enterococcus* spp. in determining CDI severity [13]. 

In this study, mice were infected with *Clostridioides difficile* following the antibiotic-mediated depletion of endogenous enterococci. The enterococcal depletion resulted in a delay in *Clostridioides difficile* colonization [13]. Afterwards, to test whether this effect was directly attributable to the enterococci in the mice gut, *Enterococcus faecalis* was introduced immediately preceding CDI. The introduction of *Enterococcus faecalis* recovered early *Clostridioides difficile* colonization [13]. 

Moreover, to test the reciprocal effect of *Clostridioides difficile* on enterococcal fitness in the gut, mice were infected with toxigenic and nontoxigenic *Clostridioides difficile* strains. Enterococcal burdens significantly increased in the presence of toxigenic *Clostridioides difficile*, demonstrating that *Clostridioides difficile* toxin-mediated damage provides a fitness advantage to *Enterococcus* spp. in the gut. 

In addition, the authors performed fluorescent in situ hybridization during CDI in mice, showing that *Enterococcus* spp. colocalize with *Clostridioides difficile* in the lumen and in biofilm-like aggregates on the host epithelium. *Clostridioides difficile* readily formed biofilms with *Enterococcus faecalis*, and this markedly enhanced *Clostridioides difficile* survival during vancomycin treatment.

Moreover, to examine the effect of *Enterococcus* spp. on CDI pathogenesis, the authors compared fecal *Clostridioides difficile* toxin titers from mice infected with *Clostridioides difficile* alone or *Clostridioides difficile* plus *Enterococcus faecalis*. *Clostridioides difficile* toxin fecal titers were higher in the mice infected with both *Clostridioides difficile* and *Enterococcus faecalis* (*p*: 0.003) [13].

Finally, the second part of the study was on pediatric human patients. During this part of the study, the authors aimed to quantify the *Enterococcus* spp. burden in pediatric patients with CDI. The authors reported a positive correlation between *Enterococcus* spp. and *Clostridioides difficile* gut burdens (Spearman’s ρ correlation: 0.551) [13].

Some authors proposed that *Enterococcus* spp. strains might have a protective effect towards *Clostridioides difficile* infections. Romyasamit et al. aimed to identify potential *Enterococcus* spp. strains exerting a protective effect against *Clostridioides difficile*. With respect to this aim, 38 fecal samples were collected from healthy breastfed infants. The authors isolated six *Enterococcus faecalis* strains inhibiting *Clostridioides difficile* spore germination and sporulation. The cytopathic effects of *Clostridioides difficile* on colon adenocarcinoma cells were reduced through a pretreatment with the cell-free supernatant of these *Enterococcus faecalis* strains [14].

### 3.3. Enterococci Intestinal Burden as a Risk Factor for CDI

The ANTICIPATE study was an international multicenter prospective observational cohort study performed to estimate CDI incidence and to assess clinical characteristics and biomarkers to predict CDI in patients receiving newly initiated antibiotic treatments [15]. The study enrolled 1007 patients receiving antibiotic treatment with penicillins, cephalosporins, carbapenems, fluoroquinolones or clindamycin. The enrolled patients were followed up for 90 days. The estimated cumulative incidence of CDI was 1.1% (95% confidence interval (CI): 0.6–2.1) and 1.9% (95% CI: 1.1–3.0) within 28 and 90 days, respectively. The study found that the high intestinal abundance of *Enterococcus* spp. relative to *Ruminococcus* spp. predicted an increased CDI risk (hazard ratio (95% CI): 5.4 (2.1–18.7)) [15]. 

The ANTICIPATE study group also investigated the intestinal microbiota of hospitalized patients to identify the microbial markers predictive of CDI. Among the 1007 patients included in the study, 135 had antibiotic-associated diarrhea, and 15 were diagnosed with CDI. Stool samples from 33 of the 135 patients with diarrhea, including 6 CDI patients were collected at the occurrence of the first diarrheal episode and were analyzed through 16S rRNA gene profiling and sequence typing. The patients developing CDI exhibited significantly lower microbial diversity prior to antibiotic treatment and a distinct microbiota phenotype enriched in *Enterococcus* spp. and depleted of *Ruminococcus, Blautia, Prevotella* and *Bifidobacterium* spp. compared to the non-CDI patients. Alpha diversity was lower in the patients developing CDI compared to the patients developing antibiotic-associated diarrhea or not developing diarrhea (*p* ≤ 0.049) [16].

Moreover, a case-control study was performed to evaluate the composition of the gut microbiota dominant bacterial groups in CDI patients compared to healthy controls. The study enrolled a total of 50 CDI inpatients and 50 healthy controls. The abundances of *Enterococcus* spp. were higher in the CDI group compared with the healthy control group (*p* < 0.05) [17].

Finally, one study used the gene sequencing approach to better understand the clinical and microbiome-based factors associated with CDI. In the study, 16S rRNA gene sequencing was used to characterize the gut microbiomes of 94 CDI patients, 89 diarrheal and 155 nondiarrheal controls. The clinical and microbiome data were merged to generate models to differentiate between the three groups of subjects. The study found that the CDI patients had a significantly higher abundance of *Enterococcus* spp. in their gut microbiota compared to the healthy controls [1]. 

### 3.4. Vancomycin-Resistant Enterococci and Clostridioides difficile

#### 3.4.1. Vancomycin-Resistant Enterococci Colonization as a Risk Factor for CDI

A retrospective cohort study was performed among nine intensive care units to evaluate the risk and pathogenic distribution of enteric infection in patients colonized with vancomycin-resistant *Enterococcus* spp. (VRE). The study included 131 VRE-colonized patients. The authors reported a trend towards an increased risk of CDI in the VRE-colonized patients. In the study, *Clostridioides difficile* was the most common pathogen detected in the VRE-colonized patients [18].

In a study performed to investigate the presence of multidrug-resistant (MDR) bacteria among hospitalized patients, stool samples from both CDI and non-CDI patients were analyzed through culture, matrix-assisted laser desorption/ionization (MALDI-TOF) mass spectrometry, polymerase chain reaction (PCR) and whole-genome sequencing for bacterial identification and characterization. The study did not find a significant difference in the prevalence of VRE between the CDI and non-CDI patients [19].

Recently, a microbiology surveillance study was performed to identify patients colonized with VRE among CDI patients admitted at a Slovakian military hospital. The authors reported that VRE was identified in 44 out of 113 (38.9%) stool samples that were positive for CD toxins [20].

#### 3.4.2. Effect of the CDI Antimicrobial Treatment on the Rate of Vancomycin-Resistant Enterococci Colonization

Oral vancomycin is one of the most frequently used antibiotics for the treatment of CDI. There are concerns that this might increase the risk of selecting vancomycin-resistant enterococci [21].

Recently, a double-blind randomized controlled trial was performed to evaluate the effect of oral vancomycin on the gut microbiota of hospitalized patients. The trial included 15 patients with at least one diarrheal stool sample that tested positive for *C. difficile* via nucleic acid amplification tests but negative via toxin enzyme immunoassay. Patients were randomized 1:1 to receive 10 days of oral vancomycin, 125 milligrams four times per day, or a matching placebo. Stool specimens were collected at 8 weeks from randomization. The authors reported an increase in microbiota beta diversity (*p*: 0.0059) in the vancomycin-treated group. Overall, VRE colonization was found in five (26%) patients, three of them in the vancomycin group. No significant difference in the prevalence of VRE was observed between the two study arms [21]. 

A multicenter retrospective study was performed to evaluate the risk of VRE among 15,780 CDI patients. The CDI patients were included if they were treated with metronidazole or oral vancomycin and had no history of VRE colonization. The authors reported no differences between the patients treated with oral vancomycin or metronidazole, developing VRE at 3 months (adjusted relative risk of 0.96; 95% CI: 0.77 to 1.20), with an absolute risk difference of −0.11% (95% CI: −0.68% to 0.47%). Similar findings were observed within 6 months [22].

A 2-year retrospective cohort study compared the VRE colonization rates between previously VRE-negative patients receiving either metronidazole or oral vancomycin as a CDI-specific treatment. In the study, 170 hospitalized CDI patients treated with metronidazole or oral vancomycin were monitored for VRE acquisition. The VRE status was assessed at the beginning of the CDI treatment in order to differentiate between the patients with and without preexisting VRE colonization. In total, 14 patients acquired VRE colonization after the first CDI antibiotic treatment. The study did not find significant differences between the VRE acquisition rates of the metronidazole- or vancomycin-treated patients (*p*: 0.98) [23].

Moreover, a large double-blind randomized multicenter phase III controlled trial was performed to test the hypothesis that fidaxomicin promotes less VRE colonization than vancomycin. In this trial, a total of 548 CDI patients were randomized to receive treatment with 10 days of fidaxomicin versus 10 days of vancomycin; of them, 301 (55%) had stool samples available both prior to and at the completion of CDI therapy: 160 vancomycin-treated patients and 141 fidaxomicin-treated patients. Pre- and posttreatment stool samples were collected and assessed for VRE colonization. In comparison with the vancomycin-treated patients, the fidaxomicin-treated patients had less frequent acquisition of VRE (8/114 patients, 7%, versus 41/133 patients, 31%; *p* < 0.001) [24].

## 4. Discussion

Currently, the exact relationship and the interplay between *Enterococcus* spp. and *Clostridioides difficile* in the human gut remain largely unknown. 

In our systematic review, we included 14 studies dealing with *Clostridioides difficile* and *Enterococcus* spp.’s interaction in the gut microbiota.

According to our results, when we looked at studies evaluating the possible role of the *Enterococci* intestinal burden as a risk factor for CDI onset, we found initial evidence that a high intestinal burden of *Enterococcus* spp. may represent a risk factor for the occurrence of CDI. On this issue, the large “ANTICIPATE” study reported that, when compared to non-CDI patients, CDI patients possess a significantly lower gut microbial diversity and a microbiota enriched in *Enterococcus* spp. [16]. 

Overall, regarding the link between VRE and *Clostridioides difficile*, the included studies did not find significant differences in the prevalence of VRE colonization between CDI and non-CDI patients. Moreover, no significant differences were reported among the included studies on the risk of developing VRE colonization after a CDI treatment course with oral vancomycin or metronidazole.

Interestingly, a large double-blind randomized phase III clinical trial reported that, in comparison with vancomycin-treated patients, fidaxomicin-treated patients had a less frequent acquisition of VRE (7% vs. 31%, *p* < 0.001) [24].

Finally, regarding the possible effect of the *Enterococci* intestinal burden in increasing CDI morbidity, studies performed in the animal model reported increased CDI severity with fecal communities enriched in *Enterococcus* spp. [12,13]. This indicated that *Enterococci* in the gut microbiota may alter the gastrointestinal environment following antibiotic treatment and support early *Clostridioides difficile* colonization and CDI occurrence.

It should be emphasized that the included studies were heterogeneous, being performed in different epidemiological settings with different designs and aims. Moreover, it is of note that, among the included studies, there are studies enrolling small study populations and therefore not reaching statistically significant results. Certainly, a major limitation of our review is the low number of included studies dealing with the specific issue regarding *Enterococcus* spp. and *C. difficile*’s interaction. 

Importantly, *Enterococcus* spp. may play a more complex role in affecting CDI pathogenesis and the outcome of CDI patients; the studies performed so far may be unable to evaluate these effects. As an example, *Enterococci* have been shown to have the potential to acquire resistance to vancomycin, which is a first-line antimicrobial agent used to treat CDI [5]. This could potentially reduce the effectiveness of vancomycin in treating *Enterococci* coinfections in CDI patients, determining worse patient outcomes.

In addition, the modulation of bacterial metabolism and the nutritional landscape in the gut microbiota may alter CDI development. A recent study suggests that *Enterococci* and *Clostridioides difficile* may interact through metabolic cross talk during CDI, enhancing reciprocal colonization, persistence and pathogenesis in the gut [13]. Again, further studies are needed to better evaluate these mechanisms.

Smith et al. recently suggested that *Enterococci* may increase *Clostridioides difficile* pathogenesis by enhancing toxin production [13]. These observations should be confirmed in studies on human subjects.

Moreover, recent studies demonstrated that *Enterococcus faecalis*’s biofilm structure is important for enhancing *Clostridioides difficile* survival following antibiotic exposure and suggested that dual-species biofilms may promote persistence during infection [13]. Importantly, biofilms provide ideal conditions for horizontal gene transfer between bacterial species. 

This preliminary evidence that *Enterococci* and *Clostridioides difficile* may share mobile genetic elements in the gut is alarming, but no studies have evaluated the potential clinical impact of the transmission of vancomycin resistance genes between VRE and *Clostridioides difficile* so far.

Interestingly, a study by Romyasamit C. et al. identified select *Enterococci* strains as potential probiotics for preventing or controlling CDI. The strains reduced the TdcA and TdcB toxic effects on human cells and prevented *Clostridioides difficile* spore production and germination [14]. This intuition may pave the road towards an innovative treatment approach against CDI, but further in vivo studies on the inhibition of *Clostridioides difficile* using ad hoc *Enterococcus* strains are required.

To conclude, the studies included in our systematic review reported evidence that the *Enterococcus* spp. intestinal burden represents a risk factor for the occurrence of CDI.

Regarding the possible link between VRE and *Clostridioides difficile*, the included studies did not report VRE colonization as a risk factor for CDI, and there is no clear evidence on the CDI treatment with oral vancomycin increasing the rate of VRE colonization. 

Overall, while the potential interplay between *Enterococcus* spp. and *Clostridioides difficile* in the human gut microbiota is still not fully understood, there is supporting evidence that *Enterococcus* spp. play a role in CDI development and clinical outcomes. Further research is needed to fully elucidate the nature of this interplay and its potential implications for CDI prevention and treatment.

## Figures and Tables

**Table 1:** Description of the studies on *Enterococcus* spp. and *Clostridioides difficile*’s interaction during CDI.

Author, Year and Country	Study Type	Setting	Study Population, Age (Mean) and Sex (% Male)	Study Aim	Study Design	Study Results
Lesniak NA et al., 2022, US [12]	Retrospective cohort study in the animal model	University germ-free mouse core laboratory	Germfree C57BL/6 mice	To test the hypothesis that specific gut bacterial communities determine variation in CDI severity.	Various gut communities were derived by colonizing germfree mice with different human fecal communities. The mice were then infected with a single *C. difficile* ribotype 027 clinical isolate.	A variation in severity was observed according to each administered human fecal community. Communities rich in *Enterococcus* spp. determined more severe CDI.
Smith AB et al., 2022, US [13]	Retrospective cohort study in the animal model and in the human model	Three university hospitals from September 2015 through December 2019	Animal model study: male mice. Retrospective cohort study: pediatric patients with median age (IQR) of 13 (5–16) years and with 50% male patients	To define the interaction between *Enterococcus* spp. and *C. difficile*. To quantify *Enterococcus* spp. burden in pediatric patients with CDI.	Mice were infected with toxigenic and nontoxigenic *C. difficile* strains following antibiotic-mediated depletion of endogenous *Enterococcus* spp. Fluorescent in situ hybridization was performed during CDI in mice. Stool samples were collected from pediatric CDI patients and were evaluated through metabolomic analyses.	*E. faecalis* burden was significantly increased in the presence of *C. difficile* toxin. *Enterococcus faecalis* colocalized with *C. difficile* in the lumen and in biofilm-like aggregates on the host epithelium. There was an enrichment of *Enterococcus* spp. in the stool of pediatric CDI patients, with positive correlation between *Enterococcus* spp. and *C. difficile* burdens (Spearman’s correlation: 0.551).
Romyasamit C et al., 2020, Thailand [14]	Microbiology study	One hospital	In total, 38 breast-fed healthy infants	To identify potential probiotic *Enterococcus* spp. strains exerting a protective effect towards CDI.	Agar well-diffusion assay was used to test the inhibitory activity of isolated colonies against toxigenic *C. difficile* strains. The cytopathic effects of *C. difficile* on colon adenocarcinoma cells were evaluated through immunofluorescence assay.	In total, 85 distinct bacterial colonies were isolated from the feces of 38 breast-fed infants. Of these, six *Enterococcus faecalis* isolates showed anti-*C. difficile* activity. The six strains inhibited spore germination (100 − 98.20 ± 2.17%) and sporulation. The cell-free supernatant of these strains reduced the cytopathic effects of *C. difficile* on colon adenocarcinoma cells (HT-29 cells).

VRE represents vancomycin-resistant *Enterococci*, CDI represents *Clostridioides difficile* infection, IQR represents interquartile range and CI represents confidence interval.

**Table 2:** Description of the studies evaluating the *Enterococcus* spp. intestinal burden as a risk factor for CDI onset.

Author, Year and Country	Study Type	Setting	Study Population, Age (Mean) and Sex (% Male)	Study Aim	Study Design	Study Results
van Werkhoven CH et al. ANTICIPATE Study Group, 2021, Europe [15]	International multicenter prospective observational cohort study	In total, 34 hospitals from France, Germany, Greece, the Netherlands, Romania and Spain from September 2016 through October 2017	In total, 1007 patients receiving newly initiated antibiotic treatment with a median age of 70 years (IQR: 62–79) and 592 (58.8%) males	To assess CDI incidence among patients receiving antibiotic treatment. To assess clinical characteristic and biomarkers to predict CDI.	Patients receiving antibiotic treatment with penicillins, cephalosporins, carbapenems, fluoroquinolones or clindamycin were followed up for 90 days. If participants reported diarrhea during the follow-up, a fecal sample was collected and tested for CDI.	The estimated cumulative incidence of CDI was 1.1% (95% CI: 0.6–2.1) within 28 days and 1.9% (95% CI: 1.1–3.0) within 90 days. High intestinal abundance of *Enterococcus* spp. relative to *Ruminococcus* spp. (hazard ratio (95% CI): 5.4 (2.1–18.7)) and low Shannon alpha diversity index (9.7 (3.2–29.7)) predicted an increased CDI risk.
Berkell M et al. ANTICIPATE study group. 2021, Europe [16]	Multicenter observational prospective study	In total, 34 hospitals from France, Germany, Greece, the Netherlands, Romania and Spain from September 2016 through October 2017	In total, 1007 patients receiving newly initiated antibiotic treatment with a median age of 70 years (IQR: 62–79) and 592 (58.8%) males. Of them, 15 were diagnosed with CDI	To identify microbial markers predictive of CDI and antibiotic-associated diarrhea.	Intestinal microbiota of the patients was investigated through 16S rRNA gene profiling combined with high-resolution sequence typing.	Patients developing CDI had significantly lower microbial diversity prior to antibiotic treatment and a microbiota enriched in *Enterococcus* spp. and depleted of *Ruminococcus, Blautia, Prevotella and Bifidobacterium* spp. Alpha diversity index was lower in patients developing CDI (*p* ≤ 0.049).
Vakili B et al., 2020, Iran [17]	Case-control study	Single hospital between 2019 and 2020	In total, 50 inpatients with CDI and 50 healthy persons	To evaluate the composition of the gut microbiota in CDI patients compared to healthy control subjects.	*C. difficile* isolates were characterized through anaerobic culture and multiplex PCR.	Higher relative abundance of *Enterococcus* spp. in the CDI group compared to the healthy control group (*p* < 0.05).
Schubert AM et al., 2014, US [1]	Retrospective cohort study	One university hospital from October 2010 to January 2012	In total, 338 individuals, including 94 CDI cases, 89 diarrheal controls and 155 nondiarrheal controls	To assess clinical and microbiome-based factors associated with CDI.	16S rRNA gene sequencing was used to characterize the gut microbiomes. Clinical and microbiome data were merged to generate models of CDI status in order to differentiate between the three groups of subjects (CDI cases and diarrheal and nondiarrheal controls).	Subjects with CDI were significantly more likely to harbor *Enterococcus* spp. in comparison to nondiarrheal controls.

PCR represents polymerase chain reaction, CDI represents *Clostridioides difficile* infection, IQR represents interquartile range and CI represents confidence interval.

**Table 3:** Description of the studies on the prevalence of vancomycin-resistant *Enterococcus* spp. in CDI patients.

Author, Year and Country	Study Type	Setting	Study Population, Age (Mean) Sex (% Male)	Study Aim	Study Design	Study Results
Axelrad JE et al., 2018, US [18]	Multicenter retrospective cohort study	Nine intensive care units within two hospitals between 2012 and 2017	In total, 716 patients. Of them, 131 were colonized with VRE, and 57 (43.5%) were male	To evaluate the risk, risk factors and pathogenic distribution of enteric infections in patients colonized with VRE.	Patients were screened for VRE colonization via rectal swab and culture. CDI was diagnosed through multiplex PCR assay.	A trend towards more CDIs was reported in patients colonized with VRE (15% vs. 10%, *p*: 0.11).
Tickler IA et al., 2020, US [19]	Microbiology study	Nine consortium laboratories between 2017 and 2018	In total, 10 CDI patients and 9 non-CDI patients, providing a total of 363 stool samples	To investigate the presence of MDR bacteria in stools from CDI and non-CDI hospitalized patients.	Stool samples were analyzed through culture, MALDI-TOF mass spectrometry, PCR testing and whole-genome sequencing.	Among 363 stool samples, 175 yielded toxigenic *C. difficile* isolates. No significant differences were observed in VRE rates between CDI and non-CDI samples.
Kuzma J et al., 2022, Czech Republic [20]	Microbiology study	One military hospital from July 2020 to September 2021	In total, 113 stool samples positive for *C. difficile* toxins	To identify VRE-colonized patients among hospitalized patients with CDI.	Stool samples were grown in a brain–heart infusion medium under aerobic conditions. The samples for VRE identification were grown on agar. The presence of vanA/vanB genes was tested through PCR.	Out of 113 samples, 44 (38.9%) harbored VRE. The most prevalent isolates were *E. faecium* (62%), *E. faecalis* (21%) and *E.* *solitarius* (9%).

VRE represents vancomycin-resistant enterococci, MDR represents multidrug resistant, MALDI-TOF represents matrix-assisted laser desorption/ionization, PCR represents polymerase chain reaction, CDI represents *Clostridioides difficile* infection, IQR represents interquartile range and CI represents confidence interval.

**Table 4:** Description of the studies on the role of CDI antimicrobial treatment in the occurrence of vancomycin-resistant *Enterococcus* spp. colonization.

Author, Year and Country	Study Type	Setting	Study Population, Age (Mean) Sex (% Male)	Study Aim	Study Design	Study Results
Fishbein SRS et al., 2021, US [21]	Double-blind randomized phase III controlled trial	One hospital from November 2017 to January 2019	In total, 15 patients with at least one diarrheal stool testing positive for *C. difficile* through PCR but negative for *C. difficile* toxins via enzyme immunoassays with a median age of 66 (37–81) and 5 males (33.3%)	To examine the effect of oral vancomycin on the gut microbiota of hospitalized patients.	Patients were randomized 1:1 to receive 10 days of oral vancomycin (125 mg, 4 times per day) versus matching placebo. Stool collection and environmental sampling were performed at enrollment, day 5, day 10, week 4 and week 8 for bacterial culturing, metagenomic analysis and whole-genome sequencing analysis.	In the vancomycin-treated group, beta diversity (*p*: 0.0059) increased after the oral vancomycin treatment. No significant difference in the prevalence of VRE colonization was observed between the two study arms.
Stevens VW et al., 2020, US [22]	Retrospective cohort study	Department of Veterans Affairs Health between 2006 and 2016	In total, 15,780 CDI patients; 5267 CDI patients treated with vancomycin alone were matched to one or more metronidazole-treated patient with a median age at CDI diagnosis of 69.0 and 94.7% male patients	To evaluate the risk of VRE following oral vancomycin or metronidazole treatment among CDI patients.	CDI patients were included if they were treated with metronidazole or oral vancomycin and had no history of VRE in the previous year. Patients were followed for VRE isolated from a clinical culture within 3 months.	Patients treated with oral vancomycin were no more likely to develop VRE within 3 months than metronidazole-treated patients (adjusted relative risk of 0.96; 95% CI: 0.77 to 1.20), and there was an absolute risk difference of −0.11% (95% CI: −0.68% to 0.47%).
Correa-Martínez CL et al., 2021, Germany [23]	Retrospective cohort study	One 1427-bed tertiary care center between 2018 and 2020	In total, 170 hospitalized CDI patients with a median age of 53 years and 94 male patients (55%)	To compare VRE colonization rates between previously VRE-negative patients receiving either metronidazole or oral vancomycin as a CDI treatment.	VRE status of CDI patients receiving treatment with metronidazole or oral vancomycin was assessed at the beginning of CDI treatment. VRE isolates collected from CDI patients were subjected to whole-genome sequencing.	In total, 14 patients (3 metronidazole-treated patients; 11 vancomycin-treated patients) acquired VRE after the CDI antimicrobial treatment. There were no significant differences in VRE acquisition rates between patients that received metronidazole and those treated with oral vancomycin (*p*: 0.98).
Nerandzic MM et al., 2012, US and Canada [24]	Double-blind randomized phase III controlled trial	Multiple hospitals	Of 548 total patients enrolled in the trial, 301 (55%) had stool samples available both prior to and at completion of CDI therapy: 160 vancomycin-treated patients and 141 fidaxomicin-treated patients	To test the hypothesis that fidaxomicin promotes VRE colonization less than vancomycin.	CDI patients were randomized to receive treatment with 10 days of fidaxomicin versus 10 days of vancomycin. Pre- and posttreatment stool samples were collected and assessed for VRE colonization.	In comparison with vancomycin-treated patients, fidaxomicin-treated patients had less frequent acquisition of VRE (8 of 114 patients (7%) vs. 41 of 133 patients (31%); *p* < 0.001).

VRE represents vancomycin-resistant enterococci, CDI represents *Clostridioides difficile* infection, MDR represents multidrug resistant, PCR represents polymerase chain reaction and CI represents confidence interval.

## Data Availability

Not applicable.

## Supplementary Materials

The following supporting information can be downloaded at https://www.mdpi.com/article/10.3390/jcm12154997/s1, Figure S1: the complete specifications of the query details used on the MEDLINE and SCOPUS databases.
