# Supplementary material for: Clostridioides difficile and Enterococci’s Interplay in the Human Gut: Bacterial Alliance or Competition? A Systematic Literature Review
Source: J Clin Med. 2023 Jul 29;12(15):4997. doi: 10.3390/jcm12154997 (PMC10420055; doi:10.3390/jcm12154997)
Supplement: Supplementary file 1 [file jcm-12-04997-s001.zip › Supplementary Figure S1.pdf]

Supplementary Figure S1. The complete specifications of the queries used to search the MEDLINE and SCOPUS database for the systematic review.

MEDLINE query:

Search: Clostridium OR clostridioides AND difficile AND Enterococcus Filters: Clinical Study, Clinical Trial, Meta-Analysis, Multicenter Study, Observational Study, Randomized Controlled Trial Sort by: First Author

(("clostridium"[MeSH Terms] OR "clostridium"[All Fields] OR ("clostridioides"[MeSH Terms] OR "clostridioides"[All Fields])) AND "difficile"[All Fields] AND ("enterococcus"[MeSH Terms] OR "enterococcus"[All Fields])) AND (clinicalstudy[Filter] OR clinicaltrial[Filter] OR meta-analysis[Filter] OR multicenterstudy[Filter] OR observationalstudy[Filter] OR randomizedcontrolledtrial[Filter])

Translations

Clostridium: "clostridium"[MeSH Terms] OR "clostridium"[All Fields]

clostridioides: "clostridioides"[MeSH Terms] OR "clostridioides"[All Fields]

Enterococcus: "enterococcus"[MeSH Terms] OR "enterococcus"[All Fields]

SCOPUS query:

clostridioides AND difficile AND enterococcus AND PUBYEAR > 2019 AND PUBYEAR < 2024 AND (LIMIT-TO (DOCTYPE , "ar")) AND (LIMIT-TO (LANGUAGE , "English")) AND (LIMIT-TO (EXACTKEYWORD , "Enterococcus"))
